# Toward reliable habitat suitability and accessibility models in an era of multiple environmental stressors

**DOI:** 10.1002/ece3.6753

**Published:** 2020-09-22

**Authors:** Hanne De Kort, Michel Baguette, Jonathan Lenoir, Virginie M. Stevens

**Affiliations:** ^1^ Plant Conservation and Population Biology Biology Department University of Leuven Leuven Belgium; ^2^ Station d'Ecologie Théorique et Expérimentale (UMR 5321 SETE) National Center for Scientific Research (CNRS) Université Toulouse III – Paul Sabatier Moulis France; ^3^ Institut de Systématique, Evolution, Biodiversité (UMR 7205) Muséum National d’Histoire Naturelle Paris France; ^4^ UR “Ecologie et Dynamique des Systèmes Anthropisés” (EDYSAN UMR 7058 CNRS‐UPJV) Université de Picardie Jules Verne Amiens Cedex 1 France

**Keywords:** Anthropocene, biological conservation, ecological niche modeling, global change, habitat suitability modeling, management, species range shifts, stacked species distribution model

## Abstract

Global biodiversity declines, largely driven by climate and land‐use changes, urge the development of transparent guidelines for effective conservation strategies. Species distribution modeling (SDM) is a widely used approach for predicting potential shifts in species distributions, which can in turn support ecological conservation where environmental change is expected to impact population and community dynamics. Improvements in SDM accuracy through incorporating intra‐ and interspecific processes have boosted the SDM field forward, but simultaneously urge harmonizing the vast array of SDM approaches into an overarching, widely adoptable, and scientifically justified SDM framework. In this review, we first discuss how climate warming and land‐use change interact to govern population dynamics and species’ distributions, depending on species’ dispersal and evolutionary abilities. We particularly emphasize that both land‐use and climate change can reduce the accessibility to suitable habitat for many species, rendering the ability of species to colonize new habitat and to exchange genetic variation a crucial yet poorly implemented component of SDM. We then unite existing methodological SDM practices that aim to increase model accuracy through accounting for multiple global change stressors, dispersal, or evolution, while shifting our focus to model feasibility. We finally propose a roadmap harmonizing model accuracy and feasibility, applicable to both common and rare species, particularly those with poor dispersal abilities. This roadmap (a) paves the way for an overarching SDM framework allowing comparison and synthesis of different SDM studies and (b) could advance SDM to a level that allows systematic integration of SDM outcomes into effective conservation plans.

## Graphical abstract

This manuscript revolves around the feasibility of dispersal‐ and evolution‐informed species distribution modeling. We provide a roadmap from data collection up to model validation to streamline species distribution modeling across taxa and levels of modeling expertise. Our roadmap demonstrates how to account for dispersal and evolution during distribution modeling of any species.

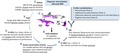

## 1 INTRODUCTION

Biodiversity is under threat across the globe, and its preservation requires transparent and effective guidelines for policy, conservation practitioners, and educators based on realistic assessments of biodiversity–environment relations (Elith & Leathwick, 2009; Kok et al., 2017; Pereira et al., 2010; Titeux, Henle, Mihoub, & Brotons, 2016). Species distribution modeling (SDM) has been a popular toolbox for studying relationships between environmental change stressors and spatial shifts in species’ suitable habitat and for predicting the potential distribution of single species, communities, and ecosystems through user‐defined environmental change scenarios (Alvarado‐Serrano & Knowles, 2014; Peterson, 2003; Weber, Stevens, Diniz‐Filho, & Grelle, 2017). The overall SDM framework is not just an interesting tool for identifying areas of local conservation concern or areas not yet occupied but potentially suitable; it has the potential to contribute substantially to the global protection of biodiversity and ecosystem services threatened by multiple environmental stressors, including land‐use change and habitat fragmentation, climate change, invasive alien species, pollution, and overexploitation (Franklin, 2013; Kok et al., 2017; Wiens, Stralberg, Jongsomjit, Howell, & Snyder, 2009).

**Figure 1:**
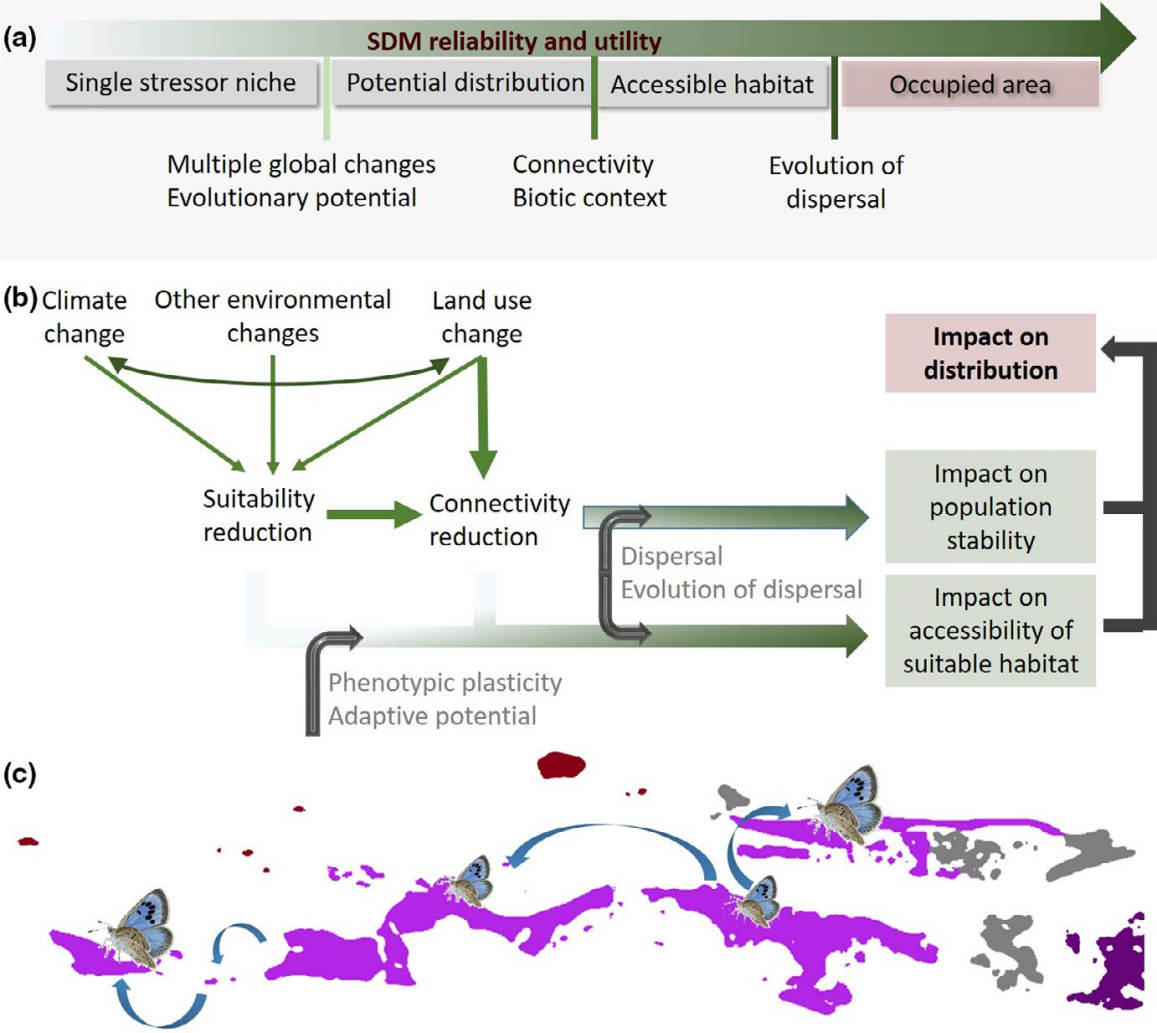
Schematic summary of the elements contributing to species distribution modeling (SDM) reliability and utility (a), the eco‐evolutionary processes underlying distribution dynamics and SDM performance (b), and a hypothetical representation of the predicted occupied distributional area of a species (*Phengaris arion*) that relies on the presence of host plants (c). Colors represent the accessible and suitable habitat (purple), habitat unsuitable due to the predicted absence of host plant (gray), habitat with host plant but inaccessible (dark purple), inaccessible habitat without host plant (dark red), and environmentally unsuitable habitat (white background). Patches occupied by larger butterflies (representing better dispersers) are predicted to be accessible due to dispersal evolution (after DeKort, Prunier, et al., 2018)

Ignoring the joint effects of multiple environmental stressors can be highly misleading; they have been found to give rise to ecological outcomes unpredicted by single environmental stressors (Bellard, Leclerc, & Courchamp, 2015; Fournier, Barbet‐Massin, Rome, & Courchamp, 2017; Guo, Lenoir, &Bonebrake, 2018; Marshall et al., 2017; Peterson & Nakazawa, 2007; Segan, Murray, & Watson, 2016; Visconti et al., 2016,) and can trigger evolutionary responses that differ from expectations assumed by single stressor evolution (Kelly, DeBiasse, Villela, Roberts, & Cecola, 2016; McClanahan, Graham, & Darling, 2014). Accounting for multiple environmental changes and potential evolutionary responses can greatly improve model accuracy (Bellard et al., 2015; Titeux et al., 2016), providing SDM outcomes that better represent the potential distribution and ultimately the occupied distributional area of the species or community under study (Figure 1). Indeed, part of the suitable habitat of the potential distribution is often not reachable due to dispersal limitation and spatially variable habitat connectivity (e.g., physical barriers), sizing the potential distribution down to the accessible habitat (Barve et al., 2011; Peterson, Papeş, & Soberón, 2015; Pulliam, 2000). Where dispersal of the focal species strongly depends on the distribution and dispersal of interacting species, the biotic context is an additional determinant of the occupied distributional area of the focal species (Figure 1). Finally, decreased connectivity among suitable habitat patches may promote evolution toward reduced dispersal (Cote et al., 2017; Graae et al., 2018), further reducing the size of the occupied distributional area (Figure 1).

In this review, we pinpoint how joint environmental changes drive population, species, and community dynamics in comparison with single stressors, focusing on climate and land‐use change as two of the most prominent threats to biodiversity. Predominantly driven by the ongoing biodiversity crisis, a universal urge for large‐scale land‐use restoration, and the existence of user‐friendly implementation tools, scientists increasingly study the impacts of both climate‐ and land‐use change on biodiversity redistribution using SDM (Araújo et al., 2019; Harrison & Gassner 2020; Milanesi, Rocca, & Robinson, 2020; Titeux et al., 2016). This ongoing SDM boost opens the door for comparing and synthesizing published SDM studies for answering taxon‐wide and large‐scale research questions, including the role of species’ traits and evolutionary potential in driving general species distribution shifts in response to land‐use change. The lack of an overarching, streamlined, and widely adoptable SDM framework containing all tools required for model preparation, parameterization, and selection, however, likely strongly refrains SDM from its full potential (Araújo et al., 2019). Moreover, a standardized and science‐based SDM toolbox would pave the way for practitioners to more systematically use SDM for conservation decision making while being aware of the potential pitfalls (Box 1). Strikingly, however, while land use has begun its debut into SDM, scientists do not generally quantify or map the amount of habitat that will likely be colonized by the species under study. To meet this concern, we specifically elaborate on the impacts of reduced habitat connectivity, a major side effect of various environmental changes, on SDM outcomes. We also investigate how dispersal is limited both by the effects of the landscape structure and configuration, and by changes in biotic interactions within communities. We then discuss why evolutionary processes should be incorporated to increase the reliability of SDM (Bush et al., 2016), and finally propose an SDM roadmap that incorporates multiple environmental stressors, dispersal, and evolution into a feasible, more reliable, and streamlined modeling framework. We stress that even for data‐insufficient systems, it is possible to incorporate the processes described above. For rare and poor‐disperser species in particular, realistic scenarios that take into account the various drivers of population and range dynamics can have crucial conservation implications. An SDM roadmap could also motivate nonexperts in the modeling field and young scientists to apply SDM to their target species, thereby facilitating the implementation of SDM in applied sciences and conservation practices. We stress, however, that expert evaluation of any SDM study is vital to a correct interpretation of SDM outcomes (see Box 1 for potential pitfalls).

Box 1 **The main sources of uncertainty that can compromise species distribution modeling (SDM)**

The main outcomes (predicted range maps) of the whole SDM framework are frequently misleading and sometimes misused (see Carlson, Chipperfield, Benito, Telford, & O’Hara, 2020) due to several main sources of uncertainty that the user, especially the beginner user, needs to have in mind when performing SDM. First, the correlative nature of SDM inherently prevents this toolbox from building on causal relationships between environmental input variables and the occurrence of a species. This *causality limitation* is unavoidable because SDM inevitably requires predictors able to reflect or capture presumed causal mechanisms from natural history knowledge. The lack of information about the causal link between the predictor variables and the response variable, usually a binary variable representing species presence–absence or presence–background data, is clearly the main limitation that the user needs to constantly keep in mind when running SDM. For instance, every single SDM method, and especially the most advanced ones such as machine learning methods, will almost always be able to find a statistical link between spatially structured predictors and the response variable used to measure species distribution. Even when predictors are known to be completely unrelated to the species distribution, such as the caricatural but very eloquent use of paintings to predict species distributions (Fourcade, Besnard, & Secondi, 2018), SDM will allow the user to draw nice‐looking maps with high prediction accuracy according to the metrics used by state‐of‐the‐art SDM methods. This suggests another important limitation hampering the use of SDM techniques which is the *misuse or overconfidence in metrics* used to measure the performance of SDM outcomes (Lobo, Jiménez‐Valverde, & Real, 2008). Indeed, typical SDM model performance metrics such as the area under the receiving operating characteristic curve (AUC), the Kappa statistic, and the true skill statistic (TSS) have been heavily criticized, as they tend to overpredict model performance under the influence of sample prevalence, consequently compromising model validation accuracy and model comparability (e.g., Leroy et al., 2018; Morán‐Ordóñez, Lahoz‐Monfort, Elith, & Wintle, 2017). Hence, the reason why these metrics are misleading measures of the performance of predictive distribution models partly relates to one last limitation to have in mind when running SDM which is the *data quality of the response variable*. Unless reliable and ground‐truth distribution data are available, such model validation parameters are extremely questionable. In situ validation of the presence or abundance data at locations for a range of predicted probability estimates therefore is highly recommended for an unbiased perspective of model performance. The final but equally important issue regarding data quality revolves around *absence data*. All the metrics used to measure the performance of SDM outcomes somehow rely on absence information, whether obtained from field observations or from a random selection strategy of background data, also known as pseudo‐absences. Clearly, collection of field‐validated true absence data across the species range is highly encouraged over pseudo‐absences selection strategies to improve SDM reliability (Leroy et al., 2018; Lobo, 2016). The sampling strategy used to select pseudo‐absences, such as considerations on the spatial extent to be used, is known to highly influence SDM outcomes (Barbet‐Massin, Jiguet, Albert, & Thuiller, 2012; Lobo, Jiménez‐Valverde, & Hortal, 2010; Van Der Wal, Shoo, Graham, & Williams, 2009). Even absence data recorded during field surveys are tricky to use and should be carefully handled depending on the aim of the undertaken SDM exercise (Hattab et al., 2017): mapping the occupied distributional area or modeling the potential distribution. If the user aims at mapping the occupied distributional area, which is more or less a spatial interpolation of the suitable and accessible locations for the focal species, then all absence data recorded during field surveys are useful for model calibration and validation: whether it represents true environmental absences or dispersal‐limited absences reflecting sites out of dispersal reach but potentially suitable regarding the abiotic and biotic conditions. In such cases, absence data bearing dispersal limitation information are very important and should be incorporated in the SDM framework together with predictor variables reflecting dispersal constraints, such as species‐specific dispersal kernel, so as to improve the mapping of the occupied distributional area (Lobo et al., 2010; Meentemeyer, Anacker, Mark, & Rizzo, 2008; Václavík & Meentemeyer, 2009). However, if the user aims at modeling the potential distribution, which implies spatial extrapolations beyond the actual occupied distributional area, then it is recommended to exclude dispersal‐limited absence from both model calibration and validation (Hattab et al., 2017). Indeed, using dispersal‐limited absences to validate the potential distribution predicted from SDM will inevitably and mathematically lead to low AUC or TSS values, which results in models misclassified as having poor predictive performance. Hence, one needs to carefully think about the meaning of the available absence data and how to use it for SDM calibration and validation steps. Modeling the potential distribution or mapping the occupied distributional area are two very different SDM exercises requiring a different thinking on the use of absence data (Hattab et al., 2017).

## 2 TOWARD ACCURATE AND MORE RELIABLE SDM

### 2.1 The climatic niche: ecological implications

The majority of SDM studies still relies on a single environmental stressor (or group of related stressors), with bioclimatic stressors (e.g., warmer and dryer conditions) representing, by far, the most popular environmental predictor used to forecast species’ distribution changes (Titeux et al., 2016). These studies observed drastic reductions in the modeled climatic suitability of currently occupied habitat for macroinvertebrates (up to 65%, Domisch et al., 2013; Parmesan et al., 1999), vertebrates (up to 80%, Warren, Wright, Seifert, & Shaffer, 2014), and plants (up to 90%, Aguirre‐Gutiérrez, van Treuren, Hoekstra, & van Hintum, 2017; Kane et al., 2017). In addition, poleward and upward shifts of species distributions are widely observed (Perry, Reid, Ibanez, Lindley, & Edwards, 2005, Kelly & Goulden, 2008, Chen, Hill, Ohlemuller, Roy, & Thomas, 2011, Lenoir & Svenning 2013) and predicted (Aguirre‐Gutiérrez et al., 2017; Barton, Irwin, Finkel, & Stock, 2016; Inoue & Berg, 2017), yet the velocity of species range shifts is generally thought to be inferior to the velocity of climate change (Bertrand et al., 2011; Chivers, Walne, & Hays, 2017; Corlett & Westcott, 2013; Devictor et al., 2012; Liang, Duveneck, Gustafson, Serra‐Diaz, & Thompson, 2017; Schloss, Nunez, & Lawler, 2012; Zhu, Woodall, & Clark, 2012).

The effective impact of climate change on biodiversity goes beyond direct climate–occurrence relations, also involving disruption of habitat connectivity and of species interactions within communities (Bertrand et al., 2016; Garcia, Cabeza, Rahbek, & Araujo, 2014; Walther et al., 2002). First, through reducing the amount of suitable habitat, climate change increases isolation between remaining patches, consequently inhibiting gene flow across the landscape and impairing population dynamics (Graae et al., 2018; Inoue & Berg, 2017; Razgour et al., 2018). Reduced gene flow increases local inbreeding risk and extinction rates, and reduces the exchange of adaptive variation (Razgour et al., 2018; Slatkin, 1987). Second, climate change renders habitat more prone to alien species’ invasions (Bellard et al., 2013; Hulme, 2017) and can alter community composition and ecosystem processes (Carroll et al., 2015; García Molinos et al., 2015; Pearson et al., 2013; Perring et al., 2016; Sheldon, Yang, & Tewksbury, 2011; Sunday, Bates, & Dulvy, 2012). Global change may therefore not only alter species’ distributions through direct abiotic environment–occurrence interactions but also indirectly through shaping the biotic context (e.g., prey, competitors, and pollinators) (Carroll et al., 2015; González‐Varo et al., 2013; Warren & Bradford, 2014; Wisz et al., 2013).

### 2.2 The potential distribution is shaped by a joint environmental niche

Over half of the terrestrial, ice‐free surface is transformed through ongoing land‐use change and habitat loss and fragmentation, considerably adding to the impacts of climate change on terrestrial biodiversity (Aguiar et al., 2016; Hansen et al., 2013; Newbold et al., 2015; Reino, Beja, Araújo, Dray, & Segurado, 2013; Titeux et al., 2016; Vitousek, 1994, but see Warren et al., 1999 for antagonistic effects). Assuming unchanged (static) land use in SDM thus renders future projections questionable (Ay, Guillemot, Martin‐StPaul, Doyen, & Leadley, 2017; Fournier et al., 2017; Perring et al., 2016; Titeux et al., 2016), yet despite the continuous recognition that land‐use change constitutes a major threat to global biodiversity (Perring et al., 2016; Titeux et al., 2016), the translation of this awareness into SDM keeps lagging behind. Given the extent and magnitude of climate and land‐use change, their combined impact on natural ecosystems is expected to be complex (Guo et al., 2018), urging for a transparent framework allowing the identification of areas susceptible to combined global change threats.

Co‐occurrence of climate and land‐use change has been shown to have interactive and often synergistic effects on biodiversity and species redistribution (Jetz, Wilcove, & Dobson, 2007; Marshall et al., 2017; Pereira et al., 2010; Visconti et al., 2016; Zwiener et al., 2017). First, land‐use change reinforces climate warming when it is associated with livestock breeding and deforestation, which considerably boost greenhouse gas emissions (Naudts et al., 2016; Reisinger & Clark, 2017). Second, land‐use change increases the amount of suitable habitat edges that are sensitive to climate change due to the absence of a protective microclimate (Brook, Sodhi, & Bradshaw, 2008; Lembrechts, Nijs, & Lenoir, 2018; Suggitt et al., 2020, Vanneste et al., 2020). Typical examples are the increased risk of forest fires due to increased wind exposure of forest remnant edges and inward desiccation of natural grasslands due to conversion of surrounding habitat into intensively managed agricultural land (e.g., Alencar, Brando, Asner, & Putz, 2015; Tuff, Tuff, & Davies, 2016). Third, deforestation can accelerate the rate of upslope range shifts in tropical regions whereas the opposite has been demonstrated for higher latitudes (Guo et al., 2018), indicating the confounding effects of land‐use change on climate‐driven range shifts. Fourth, reduced gene flow across the remaining suitable landscape jeopardizes the exchange of adaptive genetic variation that could otherwise allow evolutionary adaptation to a changing climate. Inhibited gene flow also compromises genetic diversity and fitness within remaining populations, increasing their vulnerability to environmental stressors (Frankham, Ballou, Briscoe, & Ballou, 2002; Markert et al., 2010; Schrieber & Lachmuth, 2017). A global study assessing the interactive impacts of climate and habitat loss on vertebrate diversity predicted that nearly half of the ecoregions worldwide, mainly including tropical forest, savannahs, and wetlands, will be impacted by a synergy between habitat loss and climate change during the 21st century (Segan et al., 2016).

Species distribution modeling studies increasingly include variables representing both climate and land‐use change in their models and have found varying support for land‐use change impacts on predicted species distributions, depending on the thematic resolution of the land‐use variables and the species under study (Brodie, 2016; Marshall et al., 2017; Martin, Van Dyck, Dendoncker, & Titeux, 2013; Scheller & Mladenoff, 2005; Zamora‐Gutierrez, Pearson, Green, & Jones, 2018). With the availability of a 250‐m resolution regional land‐use map and IUCN distribution maps for 286 mammal species, Brodie (2016) was able to study the interactive effects of climate change and land‐use change (through oil palm plantations) on mammal diversity in South‐East Asia. Mammal species were found to be robust to changes in climate alone, but responded dramatically to the combined effects of climate and land‐use change, with median habitat suitability losses of 47.5% (low carbon emission scenario) up to 67.7% (high emission). In a similar vein, SDM and conservation prioritization testing based on 2,255 woody plant species of the Brazilian Atlantic Forest showed that contrasting climate change scenarios did not shape conservation prioritizations, while management strategies aiming to reduce habitat fragmentation were found to be indispensable for the long‐term conservation of Atlantic Forest diversity (Zwiener et al., 2017). We stress that while the majority of SDM studies demonstrates adverse effects of climate and land‐use change on the extent of species’ distributions, a carefully implemented SDM framework could reveal the true extent of positive global change impacts on species’ distributions. For instance, forest fragmentation is likely to be detrimental to the redistribution of forest plant species—known to be poor dispersers—under climate change while it may favor the spread of more generalist plant species that are less constrained by their dispersal abilities and benefit from more open conditions inside forest edges.

### 2.3 The occupied distributional area is shaped by the accessibility of suitable habitat

The potential distribution as predicted by SDM unconstrained by dispersal limitations is fundamentally different from the occupied distributional area, that is, the distribution actually occupied by the species, requiring the integration of species’ dispersal abilities and dispersal barriers into SDM. In other words, the occupied distributional area of a given species (also referred to as the realized distribution) can be seen as a spatial interpolation of the suitable and accessible locations occupied by the focal species at a given moment in time: an instantaneous map of the real spatial occupation of the focal species. By contrast, the potential distribution can be interpreted as a spatial extrapolation, albeit not an environmental extrapolation beyond the species environmental niche, of where the species could find suitable environmental conditions to occur if it would be able to reach that location, that is, unlimited by its own dispersal abilities or by dispersal barriers.

Many studies reporting poleward or upward range shifts in the occupied distributional area also show that actual expansion rates of the studied organisms lag behind the displacement of their climatic envelopes (i.e., the potential distribution) (e.g., Bertrand et al., 2011; Bertrand et al., 2016), most likely due to dispersal and establishment lags at the leading edge (Alexander et al., 2018). Two nonexclusive factors can explain dispersal and establishment lags: (a) The displacements of individuals are slowed down by low habitat connectivity, and (b) individuals are struggling to settle viable populations in new habitat due to their dependence on biotic interactions. A basic understanding of these processes is required to assist with the implementation of dispersal‐informed SDM (Alexander et al., 2018).

#### 2.3.1 Low habitat connectivity impedes the accessibility to suitable habitat

Habitat connectivity describes how dispersal of individuals across the landscape is facilitated or impeded by landscape structure and configuration (Taylor, Fahrig, Henein, & Merriam, 1993). Dispersal, that is, movements potentially leading to gene flow among populations (Ronce, 2007), is thus key for species to track suitable habitat shifts (Berg, Julliard, & Baguette, 2010). The study of dispersal in ecology and evolution is a swiftly evolving field of investigation since almost two decades (Bowler & Benton, 2005), generating findings that are crucial for understanding the role of dispersal in SDM. Changes in landscape structure and configuration entail high dispersal costs and hence strongly affect the fitness of dispersing individuals (Bonte et al., 2012). Accordingly, theoretical models predict that dispersal will be most generally counterselected if its costs increase along habitat fragmentation gradients and exceed its expected benefits (Cote et al., 2017; Duputie & Massol, 2013; Heino & Hanski, 2001; Mathias, Kisdi, & Olivieri, 2001; Travis & Dytham, 1999). Empirical studies confirm that habitat fragmentation can decrease dispersal propensity (the probability that an individual leaves a habitat patch) and increase dispersal efficiency by reducing dispersal costs either through a reduced search time and/or through the selection of safer dispersal routes (Baguette, Blanchet, Legrand, Stevens, & Turlure, 2013; Baguette & Van Dyck, 2007). However, under particular conditions of landscape configuration and habitat suitability, theory also predicts the emergence of dispersal polymorphism within populations, in which high dispersal phenotypes with a generalist strategy coexist with low dispersal specialist phenotypes (Mathias et al., 2001). Taken together, habitat connectivity can have various ecological and evolutionary consequences, and fully ignoring this important component of the accessibility to suitable habitat may therefore strongly interfere with SDM outcomes.

To increase our understanding of the mismatch between the potential distribution (as modeled through SDM unconstrained by dispersal limitations or habitat connectivity) and the effectively occupied distributional area, the integration of dispersal and habitat connectivity metrics into SDM studies was put forward as a priority a decade ago (Araújo & Guisan, 2006; Hirzel & Le Lay, 2008). Although a growing number of studies discussed or implemented the effects of dispersal in SDM, the proportion of SDM studies implementing dispersal has remained steady for years (Bateman, Murphy, Reside, Mokany, & VanDerWal, 2013; Holloway & Miller, 2017). One of the most striking examples of the spatial lapse between potential distribution and the occupied distributional area was provided by an SDM study on Bornean Orangutans (Struebig et al., 2015). The authors predicted a loss of ca. 74% (within current range) and 84% (outside current range) of the potential Orangutan distribution by 2080s due to both climate change and direct habitat loss. However, given the sedentary lifestyle of the females, it is unlikely that the species would shift its distribution toward all suitable habitat (not all suitable habitat is accessible, even within the current range) with the predicted pace of environmental change. Hence, conservation corridors or assisted translocation is required to merge suitable and accessible distributions and to ensure long‐term persistence of this endangered species (Struebig et al., 2015). Such extreme examples of jeopardized dispersal clearly show the urgency of a paradigm shift in conservation biology distinguishing suitable from accessible. In the same vein, Albert, Rayfield, Dumitru, and Gonzalez (2017) evidenced that accounting for connectivity in spatial prioritization of protected areas for 14 focal vertebrate species strongly modified conservation priorities.

How climate change interacts with landscape features to affect dispersal is another key question, and the fine‐scale consequences of the interplay of climate and land‐use change on the spatial distribution of suitable habitat may explain dispersal lagging behind climate change (Lembrechts et al., 2018). Mestre, Risk, Mira, Beja, and Pita (2017) accordingly showed that range shift predictions consecutive to climate alterations were overly optimistic when using SDM disregarding habitat connectivity. In a very interesting study, Fordham et al. (2017) showed that models incorporating both habitat connectivity and climate suitability provided better predictions of the range shifts observed between 1970 and 2017 for 20 British bird species, than do models based on climate suitability changes alone. Such integrated modeling scenarios could be greatly simplified by translating climate changes directly into habitat connectivity changes after assessing how changes in climatic conditions modify both habitat suitability and resistance to individual movements (see also Inoue & Berg, 2017; Razgour et al., 2017). The end point of this procedure is a single model that incorporates the effects of climate change as one of the drivers of changes in habitat suitability and connectivity. Accordingly, this parsimonious approach is biologically more relevant than the production of competing models that consider the effects of either climate change or land‐use change on the accessibility of suitable habitat, whereas these two factors are clearly not independent.

#### 2.3.2 Biotic interactions modulate the probability that accessible and suitable habitat is occupied

Organisms are affected by complex networks of interactions in communities that are often neglected in SDM (Wisz et al., 2013). The higher conservation value of communities than single species is another reason for SDM to stack predictions of multiple species distributions (Guisan & Rahbek 2011). The simplest study cases are couples of species living in obligatory positive (e.g., symbiotic) or negative (e.g., parasitic) relationships. SDM targeting one partner has been shown to contribute considerably to the predictive power of the presence of its obligatory associate (e.g., DeKort, Prunier, et al., 2018; Gutiérrez, Fernández, Seymour, & Jordano, 2005; Hanspach et al., 2014). Accounting for the presence of specialist species’ partners in SDM can therefore have strong impacts on the probability that a suitable and accessible habitat is actually occupied by the focal specialist species, and usually results in more restricted species distributions as compared to SDM based on a single species (Dormann et al., 2018; Hanspach et al., 2014; Hof, Jansson, & Nilsson, 2012). In a context of shifting abiotic conditions, such close interactions can be disrupted by differences in dispersal abilities among interacting species further contributing to the observed lag in biotic responses to environmental stressors (Alexander et al., 2018). In general, effective dispersal of specialists depends on dispersal abilities of their least dispersive interactor (see also Supporting Information S1). For instance, the expansion rate of specialist butterflies to accessible and suitable habitat patches under contrasted scenarios of habitat connectivity has been shown to be severely curbed by the dispersal rates of their only host plants (DeKort, Prunier, et al., 2018; Schweiger, Settele, Kudrna, Klotz, & Kühn, 2008). Conversely, the dispersal lag between a weakly mobile parasitoid and its highly dispersive butterfly host provides the latter with enemy‐free accessible and suitable habitat patches in highly fragmented habitats (Bergerot et al., 2010).

### 2.4 Evolvability of the accessible and suitable habitat

The vast majority of SDM studies implicitly considers the relationship between an organism's ability to survive and the environmental conditions as fixed in time and space. However, the probability for a species to overcome environmental changes increases, by definition, with its adaptive potential. The ecological niche of a given species can thus evolve (Visser, 2008, Wasof et al., 2013), and local adaptation can be seen as an alternative or complementary solution for dispersal under changing environmental conditions (Graae et al., 2018). Correspondingly, phenological shifts were reported for a variety of organisms, ranging from plants (e.g., Franks, Sim, & Weis, 2007) over birds (e.g., Charmantier & Gienapp, 2014) to arthropods (e.g., Van Asch, Salis, Holleman, van Lith, & Visser, 2013). Other traits, including color morphs (Karell, Ahola, Karstinen, Valkama, & Brommer, 2011), dispersal ability (Travis et al., 2013) and the thermal niche (Rolland et al., 2018) have been shown to evolve under climate change.

The key elements to phenotypic evolution and thus the evolution of the ecological niche under climate change are local genetic additive variance (underlying interindividual variation), life history (affecting both the pace of natural evolution and the feedbacks among traits), and the interplay between dispersal and the landscape structure (balancing genetic mixing and drift). Evolutionary potential may thus differ considerably within and between species. Using a dynamic eco‐evolutionary model coupled to correlative niche projections, Cotto et al. (2017) showed that evolutionary adaptation is unlikely to prevent the predicted range contraction of long‐lived perennial alpine plant species under predicted climate change. On the contrary, evolutionary rescue was reported for insects with short generation time and fast growth (Kearney, Porter, Williams, Ritchie, & Hoffmann, 2009). Similarly, using a hybrid SDM approach that incorporates dispersal and evolutionary dynamics into correlative SDM (see Supporting information S3), Bush et al. (2016) showed that evolutionary adaptation reduces projected range losses up to 33% for 17 species of *Drosophila*. Although evolution will probably not be adequate to ensure population persistence for most species under the current pace of climate change, slowing down the pace of climate change is expected to promote evolutionary rescue (Cotto et al., 2017).

Evolution of phenotypic traits, including dispersal evolution (see Supporting information S2), thus plays a major role in shaping and conserving the potential distribution of many species.

## 3 TOWARD FEASIBLE SDM INTEGRATING LAND USE, DISPERSAL, AND EVOLUTION

### 3.1 Data collection

Researchers are increasingly motivated to share *sampling locations* on online archives, resulting in continuously growing databases. For example, the European Vegetation Archive (EVA) counted 1,529,550 vegetation plots of species co‐occurrence data most of them with geographical coordinates (Chytry et al., 2016, see also sPlot: Bruelheide, Dengler, Jiménez‐Alfaro, & Purschke, 2018a, Bruelheide et al., 2018b). In addition, the Global Biodiversity Information Facility (GBIF) currently contains 1,074,675,056 georeferenced occurrence records of microorganisms, plants, and animals. Exploring such databases for the species of interest in combination with information obtained through regional nature conservation organizations and publications can improve the detail of the spatial extent of the respective species (e.g., Cardador, Carrete, Gallardo, & Tella, 2016; Diniz‐Filho et al., 2016) (Figure 2.1). In parallel, researchers should continue to share their species survey data on publicly available repositories, while increasing computational power and data accessibility might increase the number of species for which mechanistic SDM can be parameterized. For instance, in birds, a high number of telemetry datasets were made available in public repositories such as Movebank (https://www.movebank.org) that may help parameterizing dispersal in trait‐based SDM. The integration of ecology and physiology required for trait‐based approaches will also certainly be favored by the routine integration of metabolic data into life‐history database such as Pantheria (Jones et al., 2009).

**Figure 2:**
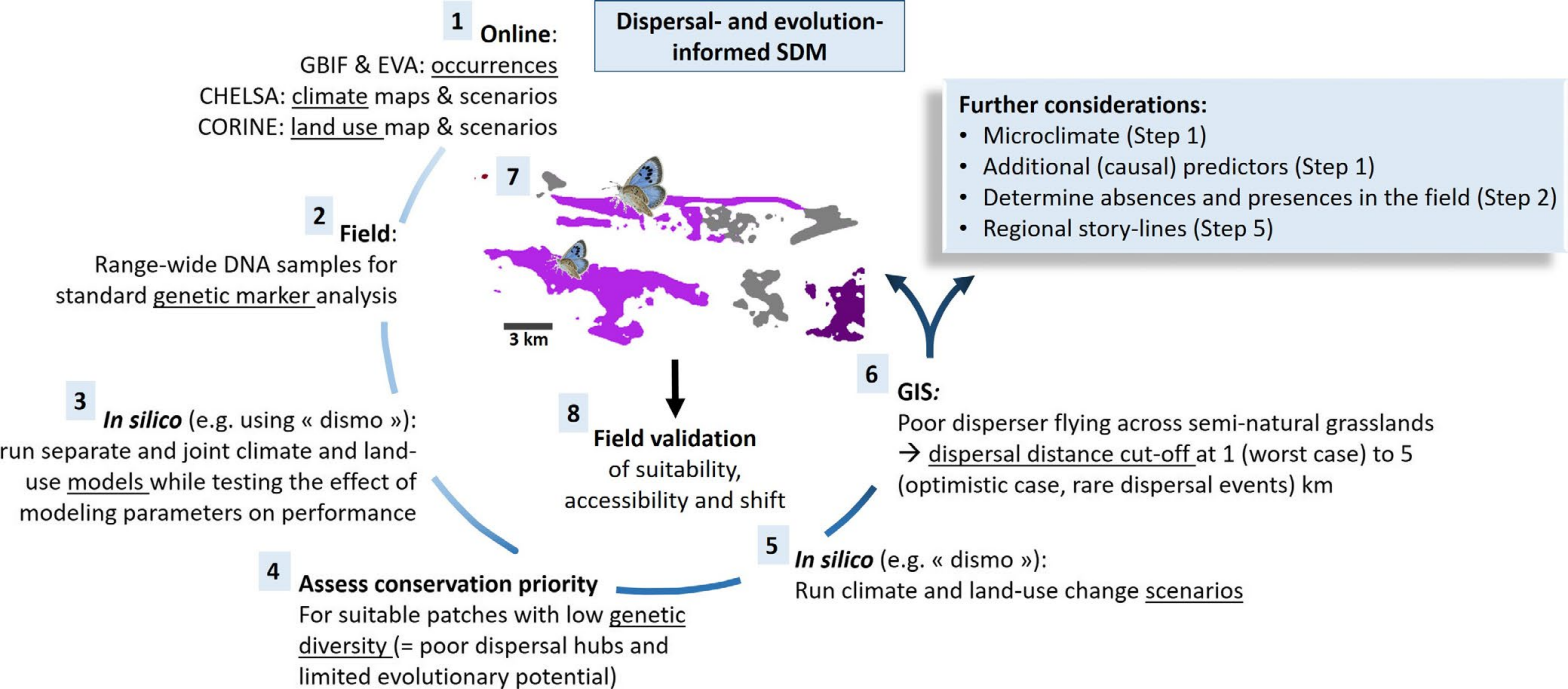
Circular roadmap presenting the necessary steps toward science‐based species distribution modeling (SDM) that is feasible for most species and implementers, with the emphasis on poor dispersers as a particularly vulnerable and valuable group of species. Note that step 4 has been given priority over step 5 because of its direct conservation implications. Step 7 represents the completion of the suitable and accessible species distribution as predicted by the SDM framework. See Figure 1 for color scheme of hypothetical distribution. We refer to the roadmap steps in the main text using bold references (e.g., Figure 2.2 refers to step 2 of the roadmap), where specific directions are provided for the respective steps. Please carefully read Box 1 to understand the importance of the “further considerations” presented in the blue rectangle of Figure 2

A struggling point complicating SDM for many species is the lack of sufficient absence and occurrence data, which is problematic because species detectability often varies in space and time, resulting in poor model performance and biased SDM outcomes (Guillera‐Arroita, 2017; Merow, Wilson, & Jetz, 2017; Morán‐Ordóñez et al., 2017; van Proosdij, Sosef, Wieringa, & Raes, 2016) (see also Box 1). To overcome *imperfect detection*, informative field surveys should be combined with a modeling approach accounting for the detection process. This could be realized through cropping the environmental input maps to the regions that were surveyed prior to extrapolation of the environment–occurrence probability relations, or through including model parameters describing the detection conditions (Acevedo, Jiménez‐Valverde, Lobo, & Real, 2012; Guillera‐Arroita, 2017; Pennino et al., 2019). The choice of absence data could furthermore have strong implications on model outcomes (Hattab et al., 2017) (see also Box 1) and are ideally categorized into: (a) environmental absence data; reflecting unsuitable habitat; (b) dispersal‐limited absences, reflecting inaccessible but suitable habitat; and where tight species’ interactions occur, (c) community‐limited absences, reflecting accessible and suitable habitat but lacking the species upon which the focal species obligatory depends. Although studies implementing field surveys that cover all types of absence data are more likely to discriminate between the potential distribution and the occupied distributional area, this distinction may be arbitrary and the selection of field‐confirmed absences in the broad sense already considerably improves model reliability (Guillera‐Arroita et al., 2015 Leroy et al., 2018) (Figure 2.2).

Environmental predictor maps (Figure 2.1) are often freely downloadable and can be merged with occurrence points using a basic GIS application. Global climate data are widely available for researchers to model past, current, and future climate projections (e.g., WorldClim and CHELSA), rendering climate niche modeling an attractive approach for global change research relative to land‐use modeling. Yet, also regional and global land‐use maps have become accessible (e.g., GlobCover, MODIS2005, CORINE) and can be converted from a vector to an SDM‐friendly raster format using any geographical information system (GIS rasterizing) to model land‐use and habitat connectivity. Moreover, while the short‐term nature of land‐use maps and land‐use change may complicate land‐use change SDM, simple land‐use change scenarios could be tested in a sensitivity analysis for a given near‐future climate change scenario. Where land cover maps generally lack resolutions beyond the dominant land cover types (e.g., “grasslands” and “urban area”), there are several ways that allow more ecologically relevant land‐use mapping. Information on the protection level of areas across the globe (e.g., through the protected area network) (Kremen et al., 2008) can be merged with a land cover map to indicate the level of management of specific land cover types (Rodrigues et al., 2004). Grasslands and forests are, for example, more likely to be intensively managed or exploited when unprotected. Alternatively, human population density maps and road maps can be used to fine‐tune the intensity of land disturbance (e.g., Newbold et al., 2015), while a forest cover map can be integrated to model high‐resolution forest change for species strongly depending on the absence or presence of forests (e.g., Hansen et al., 2013). Finally, inclusion of microclimatic variables such as topography not only allows increasing the spatial resolution and accuracy of SDM predictions, it also has been demonstrated to play an underappreciated role in shaping the trajectories of species evolution and redistribution (De Kort et al., 2020; Suggitt et al., 2020).

The choice of environmental predictors is crucial for SDM, as noncausal and redundant predictors unnecessarily increase model complexity and frequently give rise to misleading model performance estimates and flawed projections (Brodie et al., 2020; Fourcade et al., 2018; Warren, Matzke, & Iglesisa, 2020). Therefore, SDM should only be used when the presumed causality and ecological relevance of tested predictors are carefully considered (Figure 2.1) (see also Box 1). When soil water availability, for example, has been suggested to be deterministic for the presence of the species under study, a wetness index map or equivalent, should evidently be incorporated during modeling.

### 3.2 Model parameterization and in silico validation

Although the technical aspects underlying model evaluation lie beyond the scope of this review (see, e.g., Aiello‐Lammens, Boria, Radosavljevic, Vilela, & Anderson, 2015; Muscarella, Galante, Soley‐Guardia, & Boria, 2014; Peterson et al., 2007; Phillips & Dudík, 2008; Radosavljevic & Anderson, 2014, for techniclal SDM considerations), we highlight the importance of model tuning and parameterization as a key criterion for realistic modeling (Box 1). The predictive performance of SDMs is usually evaluated through cross‐validation, using training data to fit models and a set of testing data that is spatially independent of the training data to evaluate these models (Hijmans, 2012). This model evaluation approach is, however, highly sensitive to spatial autocorrelation of environmental variables between training and testing data, and only tests the set of known occurrence data. These drawbacks artificially inflate cross‐validated model statistics (Hijmans, 2012; Morán‐Ordóñez et al., 2017) and stress the importance of in situ validation (see below).

We recommend SDM users to consistently model at least three scenarios: climate only, land use only, and climate and land use together, in addition to a full model that incorporates potential additional predictors such as soil moisture and biotic interactions (Figure 2.3). Such a standardized SDM framework allows (a) addressing model complexity issues (see Brun et al., 2020; Gregr, Palacios, Thompson, & Chan, 2019) and (b) comparing the contribution of major environmental change stressors to predicted shifts in species distributions between independent SDM studies. Although more efforts are needed for true cross‐species and cross‐study comparison of SDM outcomes (e.g., the development of an overarching, decision tree‐based SDM environment that systematically informs users about parameter and predictor choice and more detailed land‐use scenarios), we emphasize the promise that standardizing SDM holds (a for uncovering overall effects of dispersal, evolution, life history, and anthropogenic stressors on the projected distribution of many species and communities, and (b) for framing each study into a much larger and significant context.

### 3.3 Integrate evolutionary potential

Integrating *evolution* into SDM without assumptions on mutation and demographic rates (Supporting information S3), which can be particularly challenging for rare species, can be achieved if the evolutionary potential of populations across the study area is quantified (Gotelli & Stanton‐Geddes, 2015; Ikeda et al., 2017). Quantitative genetic screening of phenotypic traits has long been thought to provide the most accurate information on the evolutionary potential of traits and life‐history syndromes. Such quantitative genetic research is now, however, questioned due to drawbacks related to limited statistical power, high time consumption, unrealistic assumptions on the genetic architecture underlying adaptive traits and poor representation of natural conditions (Hoffmann, Sgrò, & Kristensen, 2017; Wood, Yates, & Fraser, 2016). More recently, the use of genomic markers representing neutral and/or adaptive genetic variation has been proposed and tested for modeling local adaptation and adaptive potential in SDM (Fitzpatrick & Keller, 2015; Ikeda et al., 2017; Marcer, Méndez‐Vigo, Alonso‐Blanco, & Picó, 2016). As a consequence of local adaptation, many populations develop a local genetic signature shaped by historical and current environmental conditions (Watanabe & Monaghan, 2017). Variation in population responses and environmental change is thus expected, and shown, to affect the ability of SDMs that do not incorporate genetic structure to predict future species’ distributions (Ikeda et al., 2017; Marcer et al., 2016).

In addition to the implementation of neutral genetic structure, explicitly incorporating genetic variation underlying adaptive traits can be achieved through ecology‐informed genome screening (e.g., De Kort & Honnay, 2017). As phenotypic traits respond to environmental change through shifts in the underlying genes, associations between genetic and environmental variation are assumed to result from local adaptation (Manel, Schwartz, Luikart, & Taberlet, 2003; Storfer et al., 2007). Even without a sequenced reference genome, landscape genomic analysis of genetic markers, allowing the identification of genetic patterns associated with environmental variation (e.g., climate or land use), is a feasible strategy for any species (Frichot, Schoville, Bouchard, & François, 2013; Manel & Holderegger, 2013; Rellstab, Gugerli, Eckert, Hancock, & Holderegger, 2015; Sork et al., 2016). High variation at genetic variants associated with temperature or habitat fragmentation may therefore indicate a high potential to adapt to future climate and land‐use change. While the integration of this *adaptive potential* into SDM is under development (e.g., Peterson, Doak, & Morris, 2019), simple correlations between neutral and adaptive genetic diversity on the one hand, and SDM habitat suitability estimates on the other hand, indicates the extent to which adaptive evolution may affect SDM projections. High adaptive potential at the rear edge, for example, may prevent local extinctions despite a projected northward shift with climate change (Erichsen et al., 2018; Exposito‐Alonso et al., 2018). Low genetic diversity at suitable locations, on the other hand, may be indicative of imminent local extinction and poor connectivity, and/or of relatively recent colonization after a period of reduced habitat suitability (e.g., Gutiérrez‐Rodríguez, Barbosa, & Martínez‐Solano, 2017; DeKort, Baguette, et al., 2018; DeKort, Prunier, et al., 2018). Conservation actions focusing on expanding or connecting SDM‐based suitable patches holding populations of low genetic diversity may consequently increase options for dispersal and evolution. We therefore recommend conservation prioritization based on a combination of SDM and genetic marker assessment (Figures 2.2 and 2.4). Although the collection of genetic material for genetic marker analysis may be particularly challenging for rare species, noninvasive sampling such as fecal, hair, and eggshell sampling may overcome this issue (e.g., Beja‐Pereira, Oliveira, Alves, Schwartz, & Luikart, 2009) (Figure 2.2).

### 3.4 Run global change scenarios

Depending on the scale of the study, *land‐use change scenarios* (Figure 2.5) could rely on local managers that are aware of ongoing land‐use developments, regional storylines related to demands of arable production, livestock number, urbanization, and/or international socioeconomic parameters (e.g., DeKort, Prunier, et al., 2018; Dullinger et al., 2020; La Sorte et al., 2017; Marshall et al., 2018), or global land‐use scenarios as outlined by the Intergovernmental Panel on Climate Change (IPCC). Land‐use variables can subsequently be manipulated (e.g., partial conversion of extensive grasslands into forest, or forest into built‐up area) using basic GIS applications to generate SDM scenarios integrating both climate change and habitat fragmentation (e.g., Lehsten et al., 2015; Marshall et al., 2018; Martin et al., 2013). *Climate change scenarios* have been outlined by the IPCC and are freely available at chelsa‐climate.org and WorldClim.org.

At this point, all data are available for modeling, using *dismo* or another SDM framework (Figure 2.5). Models are first trained and tested using the occurrence points and (cropped) environmental maps, finally providing a habitat suitability map that reflects the present distribution. The model results are then extrapolated to predict future distributions under the provided scenarios. These projections do not account for dispersal and evolution, and require fine‐tuning and contextualization based on species’ life‐history traits (through partial dispersal modeling, Figure 2.6) and genetic markers (through discussion of the adaptive potential, Figure 2.4).

### 3.5 Integrate dispersal into SDM

Dispersal‐informed SDM evidently requires a basic understanding of *dispersal behavior* in the species under study. Obtaining this information could be particularly challenging for species that are hard to monitor or for plant species that depend on vectors for seed dispersal. For plants, dispersal syndrome (wind, animal, ant, and ballistic or no syndrome) and growth form (tree, shrub, and herb) provide reasonable predictions of maximum dispersal distance (MacLean & Beissinger, 2017; Tamme et al., 2014; Thomson, Letten, Tamme, Edwards, & Moles, 2018), allowing integration of dispersal into SDM through simple field observations (e.g., Midgley, Hughes, Thuiller, & Rebelo, 2006; Peyre et al., 2020). For animals, movement ability, longevity, and habitat breadth are important predictors of dispersal distance and climate change‐induced range shifts (MacLean & Beissinger, 2017; Stevens et al., 2013). These *life‐history traits* can therefore be considered to define a partial dispersal SDM approximating the occupied distributional area to a more accurate extent than a SDM assuming no dispersal (Bateman et al., 2013). Alternatively, it is possible to create a species‐specific dispersal kernel and use it as a predictor variable capturing the impact of dispersal limitations on the occupied distributional area (Hattab et al., 2017; Meentemeyer et al., 2008; Václavík & Meentemeyer, 2009) (see Box 1). Even relatively simple partial dispersal models, where the potential distribution has been clipped down to accessible distribution based on estimates of maximum dispersal distances, have been shown to improve distribution projections under environmental change (DeKort, Prunier, et al., 2018; Fitzpatrick, Gove, Sanders, & Dunn, 2008; Meier et al., 2012; Midgley et al., 2006). The implementation comfort and the limited number of assumptions related to demographic rates make this type of partial‐dispersal SDM the preferred option for many species (Bateman et al., 2013). The most reliable and informative partial dispersal SDMs are expected for species with poor dispersal capacities, because *poor dispersers* (a) are often of high conservation concern, (b) facilitate integration of dispersal into SDM through assuming limited dispersal between suitable patches (Figure 2.6), and (c) provide conservative estimates of patch accessibility for associated species. Although we do not specifically recommend to focus on poor dispersers, we do believe that this important target group should receive particular attention in future SDM studies aiming to develop dispersal‐ and evolution‐informed conservation strategies. Although model accuracy improves considerably in partial dispersal SDM, they still not fully reflect real conditions. A more mechanistic approach, for example, through hybrid models integrating both correlative and mechanistic principles, could further increase model reliability (see Supporting information S3).

### 3.6 In situ model validation

In silico model parameterization and validation should be complemented with in situ model evaluation in unsampled regions, through extracting a set of suitable and unsuitable habitat coordinates from model output and empirically evaluate occurrence in the field (Araujo, Pearson, Thuiller, & Erhard, 2005; DeKort, Prunier, et al., 2018) (Figure 2.8). Among the rare examples of studies using in situ SDM validation, Williams et al. (2009) were able to find 24 new localities (out of 36 checkpoint sites) shared among four rare plant species across the Rattlesnake Creek Terrane in California. Area under the curve (AUC) of the receiver operating characteristic (Hanley & McNeil, 1982), a commonly used model validation statistic retrieved from the cross‐validation approach, ranged between 0.94 and 0.98, commonly interpreted as nearly perfect predictive performance (but see Box 1 for pitfalls related to this model validation metrics like the AUC statistic). Two important conclusions can be drawn from this study. First, there can be considerable inconsistency between in silico (simulated) and in situ (real) model validation, which may reflect (a) the drawbacks of in silico model validation methods and (b) the (in)accessibility of suitable habitat patches. Second, SDM studies can be highly suitable for conservation purposes, given that (a) validation was performed both in silico using independent calibration and testing data and in situ, (b) relevant environmental maps and scenarios are generated, and (c) dispersal and evolution are implemented. We finally recommend projected shifts in distributions to be followed up in situ at regular time intervals (Figure 2.7) (see, e.g., Areias Guerreiro, Mira, & Barbosa, 2016; Barbet‐Massin, Rome, Villemant, & Courchamp, 2018; West, Kumar, Brown, Stohlgren, & Bromberg, 2016). This has two major advantages, including the ability to test the power of SDM approaches to predict distribution shifts and to assess the impact of conservation actions on projected shifts in SDM.

## CONFLICT OF INTEREST

No competing interests.

## AUTHOR CONTRIBUTION

**Hanne De Kort:** Conceptualization (equal); Writing‐original draft (lead); Writing‐review & editing (equal). **Michel Baguette:** Conceptualization (equal); Funding acquisition (lead); Writing‐original draft (equal); Writing‐review & editing (equal). **Jonathan Lenoir:** Conceptualization (supporting); Supervision (equal); Writing‐review & editing (equal). **Virginie Stevens:** Supervision (lead); Writing‐review & editing (equal).

## Supporting information

Supinfo

## Data Availability

All data used were accessed through referenced literature.
