# Supplementary material for: Toward reliable habitat suitability and accessibility models in an era of multiple environmental stressors
Source: Ecol Evol. 2020 Sep 22;10(20):10937–52. doi: 10.1002/ece3.6753 (PMC7593202; doi:10.1002/ece3.6753)
Supplement: Supplementary file 1 — Supinfo [file ECE3-10-10937-s001.docx]

**Supporting Information files.**

SI 1. Effect of environmental change on species networks and dispersal capacities

SI 2. Effect of environmental change on the evolution of dispersal traits

SI 3. An introduction to correlative vs. mechanistic modelling

SI 3.1. Mechanistic dispersal modeling

SI 3.2. Mechanistic evolution modeling

**SI 1. Effect of environmental change on species networks and dispersal capacities**

In a changing environment, focussing on changes in larger communities rather than on couples of closely interacting species could be a much more complicated exercise: both the variety and the quantity of interspecific interactions to take into account are tremendously increasing (Wisz *et al.* 2013; Anderson 2017). A recent theoretical exploration of how networks of interacting species will react to environmental changes emphasised the pivotal role of dispersal in changing species interactions within accessible and suitable habitat patches and in creating new species assemblages (Thompson & Gonzalez 2017). When dispersal rates are low, all types of interactions are strong determinants of suitable habitat colonization, which eventually leads to the formation of new networks of interacting species. When dispersal rates are high, a higher proportion of the network can invade existing communities within accessible and suitable habitat patches, which lowers the formation of new assemblages and increases the risk of biotic homogenization. In general, the integrity of the biotic relationships between species thus depends on the dispersal ability of the least dispersive interactor. The encouraging conclusion here is a large parallelism of the crucial role of dispersal between couples of interacting species and more complex species networks. However, experimental and/or empirical validations of the predictions of multi-species dispersal models are a top priority in the research agenda of SDM research, where it presents considerable practical difficulties, as knowing why, where and how individuals of interacting species disperse remains a black box (e.g. Baguette et al. 2014). To bypass this apparent idiosyncrasy of the dispersal process, one possibility could be to rely on general relationships between dispersal and life-histories. Dispersal interacts with many life-history traits, leading to trade-offs referred to as dispersal syndromes: species with contrasted dispersal capacities have well defined suites of demographic, physiological, behavioural or morphological traits (Ronce & Clobert 2012). The life-histories of interacting species within a network could thus provide the opportunity to predict their dispersal distances (Stevens et al. 2013). The exploration of this promising research avenue could greatly facilitate metacommunity ecology and SDM.

**SI 2. Effect of environmental change on the evolution of dispersal traits**

Dispersal evolution is a requirement for adjusting range shifts in the face of global change. Meta-analyses report that dispersal shows a high intra-specific variability (Stevens *et al.* 2010b, a). In line with this variability, the emergence of coexisting dispersal strategies under particular conditions of habitat suitability and accessibility has been predicted by theoretical models (Travis & Dytham 1999, Mathias 2001, Bonte et al. 2010) and confirmed empirically (Baguette et al. 2012; Baguette et al. 2013). This strong dependence of dispersal on the environmental context suggests rapid evolution of dispersal abilities at the intra-specific level in response to external stressors including habitat fragmentation (Baguette et al. 2012) and climate change (Le Gaillard et al. 2012). Moreover, as previously mentioned, dispersal is strongly linked to other life history traits through dispersal syndromes that affect the efficacy of dispersal evolution, as super-disperser strategies (e.g. combining increased fecundity and movement) are highly beneficial in the process of suitable habitat colonization (Ronce & Clobert 2012, Stevens et al. 2013, 2014, Legrand et al. 2016, Cote et al. 2017). The evolution of dispersal syndromes has been predicted by theoretic studies (Bocedi *et al.* 2014b; Cote *et al.* 2017), and empirically demonstrated in the invasive yellow-bellied toad (Cayuela et al. 2016,). The extinction of whole life-history tactics (notably those associated with a resident strategy) may constitute a silent loss of biodiversity of which we absolutely do not measure the possible consequences on the functioning of populations, of the communities they constitute with their interactors, and of the ecosystems they belong to.

**SI 3. An introduction to correlative vs. mechanistic modelling**

Two families of SDM approaches, i.e. correlative and mechanistic models, have been developed to address the relationship between species occurrences and absences, and the associated environmental conditions. Correlative species distribution modelling (SDM) (e.g. Guisan and Zimmermann 2000) utilize the observed relationship between species presence and environmental conditions (biotic and/or abiotic) (see a review in Kissling et al. 2012). Observational data for correlative SDM approaches are typically gathered over wide spatial scales. The correlative approach, as implemented in the popular MAXENT software (Phillips and Dudík 2008, Merow et al. 2013), is by far the most commonly used approach for SDM studies (e.g. Carvalho et al. 2017) and it allows quick predictions even when the underlying processes driving species distributions are unknown. Unfortunately, the correlative nature of SDM inherently limits their reliability in forecasting species distribution under new environmental conditions (Araújo & Guisan 2006). For this reason, correlative SDM approaches are perfect for interpolating species distribution within the region of calibration and during the period of calibration but such models perform poorly to extrapolate species distribution outside the range of conditions used for calibration. Additionally, using simulation and field data, Ashcroft et al. (2017) evidenced that fatal reductions in population density may occur long before any change in local species occurrence and distribution range can be detected. As a consequence, although they can easily be parameterized for many species, correlative SDM based on presence (or presence/absence) data may provide over-optimistic forecasts of species persistence under climate change.

Contrary to correlative niche models, mechanistic models study the effect of environmental variables (both biotic and abiotic) on individual or population performances (Cabral & Kreft 2012). These mechanistic models integrate functional traits (e.g. dispersal, physiology, competitive ability) and fitness components (e.g. fecundity, survival, growth) to fine-tune eco-evolutionary processes. The parameterization of mechanistic models can be much harder to achieve as they usually require specific, fine-scale data (Dormann *et al.* 2012), especially when considering the possibility for local adaptations that may change the relationship between environmental variables and individual or population performances through space or time. Mechanistic approaches are however in solving several issues identified here. For instance, an individual-based model that considered dispersal limitations, competition and facilitation was successfully used to estimate the altitudinal shift of the tree line in response to climate change in the Pyrenees (Martínez *et al.* 2012).

Both SDM approaches have their own advantages and limitations, reflecting two extremes of a continuum between higher realism associated with huge data demand, and lower realism obtained with simpler data (see e.g. Hijmans and Graham 2006, Gutt et al. 2012, Estes et al. 2013, Torossian et al. 2016). Given the relative advantages and divergent results (e.g. Shabani et al. 2016) provided by both approaches, several authors argued for the use of (i) hierarchical approaches combining correlative approaches at large spatial or temporal scales and mechanistic models at smaller scales, or (ii) hybrid modelling approaches (Dullinger et al.2012, Torossian et al. 2016, Zurell et al. 2016) incorporating both methods. Briscoe et al. (2016), for instance, linked a correlative to a mechanistic model to forecast the use of refugia by koalas under climate change scenarios. Using both simulated and empirical datasets, Talluto et al. (2016) finally demonstrated how model uncertainty decreased with such an integrated approach.

**SI 3.1. Mechanistic dispersal modeling**

Despite decisive advances in forecasting species distributions, the integration of dispersal variability and evolutionary changes in the mechanistic part of hybrid niche models often remains an issue. In a thorough review presenting how movements can be incorporated into species distribution models, Miller and Holloway (2015) comprehensively detailed the various approaches used to predict the accessible set of suitable habitat patches. They identified several steps from simple constant diffusion rates of species across landscapes to complex population dynamics models, through the use of dispersal kernels and landscape metrics. An additional difficulty comes from the need to upscale patterns observed, and processes acting at, finer spatial scales to provide predictions of range shifts at larger scales (Bocedi *et al.* 2014a). RangeShifter (Bocedi et al. 2014a) is an individual-based modeling platform that incorporates several aspects of population dynamics, as well as inter-individual variability and dispersal details, to predict large-scale range shifts. Combining similar individual-based models to correlative species distribution models should allow for identifying the processes as well as the locations likely to be key for species persistence under climate change, while providing a realistic view of the uncertainty of the forecasts. Such integration was recently proposed by Cotto et al. (2017), who sequentially applied a correlative model and an eco-evolutionary model to four alpine plant species. Their results show how correlative species distribution models alone, ignoring local evolutionary and demographic processes, returned over-optimistic forecasts.

**SI 3.2. Mechanistic evolution modeling**

Evolution is highly trait-, species- and context-dependent, requiring knowledge of, or strong assumptions on, mutation, survival, extinction and gene flow rates in evolution-informed SDM, and is therefore typically modelled using a mechanistic approach (Bocedi et al. 2014, Cotto et al. 2017). Alternatively, Bush et al. (2016) developed a hybrid SDM approach allowing users to define parameters of adaptive capacity and dispersal probability, using the R package “AdaptR”. Trait-based modeling procedure appears as another promising SDM tool accessible for many species. This special case of mechanistic modeling utilizes the conservation laws of thermodynamics describing energy and mass balance at the level of individuals to link functional traits (morphology, physiology, or behaviour) to characteristics of the environment. These relationships are subsequently used to calculate activity constraints, demographic traits (survival, growth, reproduction), and ultimately species distribution. The example of a trait-based SDM applied to cane toad expansion in Australia (Kearney et al. 2008) shows that the required information is relatively simple to gather either from existing physiological data on similar organisms (in that case other amphibians) or from simple, targeted experiments or field surveys. Trait-based SDM appear promising in terms of accuracy, and hence explanatory and predictive power because they: (i) make direct use of the fitness effect of the relationship between functional traits and variables of the physical environment; (ii) consider nutrition, thereby capturing the essence of a primary driver of species interactions; (iii) allow intra-specific variability in the traits considered, be it due to adaptation or other evolutionary processes (Kearney et al. 2010, Benito Garzón et al. 2019); and (iv) allow the direct modeling of trait associations, notably dispersal-associated syndromes of traits as exemplified by Moor (2017).

**References**

Anderson, R.P. (2017). When and how should biotic interactions be considered in models of species niches and distributions? *J. Biogeogr.*, 44, 8–17.

Araújo, M.B. & Guisan, A. (2006). Five (or so) challenges for species distribution modelling. *J. Biogeogr.*, 33, 1677–1688.

Ashcroft, M.B., King, D.H., Raymond, B., Turnbull, J.D., Wasley, J. & Robinson, S.A. (2017). Moving beyond presence and absence when examining changes in species distributions. *Glob. Chang. Biol.*, 23, 2929–2940.

Bocedi, G., Palmer, S.C.F., Pe’er, G., Heikkinen, R.K., Matsinos, Y.G., Watts, K., *et al.* (2014a). RangeShifter: a platform for modelling spatial eco-evolutionary dynamics and species’ responses to environmental changes. *Methods Ecol. Evol.*, 5, 388–396.

Bocedi, G., Zurell, D., Reineking, B. & Travis, J.M.J. (2014b). Mechanistic modelling of animal dispersal offers new insights into range expansion dynamics across fragmented landscapes. *Ecography (Cop.).*, 37, 1240–1253.

Briscoe, N.J., Kearney, M.R., Taylor, C.A. & Wintle, B.A. (2016). Unpacking the mechanisms captured by a correlative species distribution model to improve predictions of climate refugia. *Glob. Chang. Biol.*, 22, 2425–2439.

Bush, A., Mokany, K., Catullo, R., Hoffmann, A., Kellermann, V., Sgrò, C., *et al.* (2016). Incorporating evolutionary adaptation in species distribution modelling reduces projected vulnerability to climate change. *Ecol. Lett.*, 19, 1468–1478.

Cabral, J.S. & Kreft, H. (2012). Linking ecological niche, community ecology and biogeography: Insights from a mechanistic niche model. *J. Biogeogr.*, 39, 2212–2224.

Carvalho, B.M., Rangel, E.F. & Vale, M.M. (2017). Evaluation of the impacts of climate change on disease vectors through ecological niche modelling. *Bull. Entomol. Res.*, 107, 419–430.

Cayuela, H., Boualit, L., Arsovski, D., Bonnaire, E., Pichenot, J., Bellec, A., *et al.* (2016). Does habitat unpredictability promote the evolution of a colonizer syndrome in amphibian metapopulations? *Ecology*, 97, 2658–2670.

Cote, J., Bestion, E., Jacob, S., Travis, J., Legrand, D. & Baguette, M. (2017). Evolution of dispersal strategies and dispersal syndromes in fragmented landscapes. *Ecography (Cop.).*, 40, 56–73.

Dormann, C.F., Schymanski, S.J., Cabral, J., Chuine, I., Graham, C., Hartig, F., *et al.* (2012). Correlation and process in species distribution models: Bridging a dichotomy. *J. Biogeogr.*, 39, 2119–2131.

Estes, L.D., Bradley, B.A., Beukes, H., Hole, D.G., Lau, M., Oppenheimer, M.G., *et al.* (2013). Comparing mechanistic and empirical model projections of crop suitability and productivity: Implications for ecological forecasting. *Glob. Ecol. Biogeogr.*, 22, 1007–1018.

Guisan, A. & Zimmermann, N.E. (2000). Predictive habitat distribution models in ecology. *Ecol. Modell.*, 135, 147–186.

Gutt, J., Zurell, D., Bracegridle, T.J., Cheung, W., Clark, M.S., Convey, P., *et al.* (2012). Correlative and dynamic species distribution modelling for ecological predictions in the Antarctic: A cross-disciplinary concept. *Polar Res.*, 31.

Hijmans, R.J. & Graham, C.H. (2006). The ability of climate envelope models to predict the effect of climate change on species distributions. *Glob. Chang. Biol.*, 12, 2272–2281.

Kearney, M., Simpson, S.J., Raubenheimer, D. & Helmuth, B. (2010). Modelling the ecological niche from functional traits. *Philos. Trans. R. Soc. B Biol. Sci.*, 365, 3469–3483.

Kissling, W.D., Dormann, C.F., Groeneveld, J., Hickler, T., Kühn, I., Mcinerny, G.J., *et al.* (2012). Towards novel approaches to modelling biotic interactions in multispecies assemblages at large spatial extents. *J. Biogeogr.*, 39, 2163–2178.

Legrand, D., Larranaga, N., Bertrand, R., Ducatez, S., Calvez, O., Stevens, V.M., *et al.* (2016). Evolution of a butterfly dispersal syndrome. *Proceedings. Biol. Sci.*, 283, 20161533.

Martínez, I., González-Taboada, F., Wiegand, T., Camarero, J.J. & Gutiérrez, E. (2012). Dispersal limitation and spatial scale affect model based projections of Pinus uncinata response to climate change in the Pyrenees. *Glob. Chang. Biol.*, 18, 1714–1724.

Merow, C., Smith, M.J. & Silander, J.A. (2013). A practical guide to MaxEnt for modeling species’ distributions: what it does, and why inputs and settings matter. *Ecography (Cop.).*, 36, 1058–1069.

Miller, J.A. & Holloway, P. (2015). Incorporating movement in species distribution models. *Prog. Phys. Geogr.*, 39, 837–849.

Moor, H. (2017). Life history trade-off moderates model predictions of diversity loss from climate change. *PLoS One*, 12, 1–21.

Phillips, S.J. & Dudík, M. (2008). Modeling of species distributions with Maxent: new extensions and a comprehensive evaluation. *Ecography (Cop.).*, 31, 161–175.

Shabani, F., Kumar, L. & Ahmadi, M. (2016). A comparison of absolute performance of different correlative and mechanistic species distribution models in an independent area. *Ecol. Evol.*, 6, 5973–5986.

Stevens, V.M., Pavoine, S. & Baguette, M. (2010a). Variation within and between Closely Related Species Uncovers High Intra-Specific Variability in Dispersal. *PLoS One*, 5, e11123.

Stevens, V.M., Trochet, A., Blanchet, S., Moulherat, S., Clobert, J. & Baguette, M. (2013). Dispersal syndromes and the use of life-histories to predict dispersal. *Evol. Appl.*, 6, 630–642.

Stevens, V.M., Turlure, C. & Baguette, M. (2010b). A meta-analysis of dispersal in butterflies. *Biol. Rev.*, 85, no-no.

Storey, J., Bass, A., Dabney, A. & Robinson, D. (2019). qvalue: Q-value estimation for false discovery rate control. R package version 2.14.1,.

Talluto, M. V., Boulangeat, I., Ameztegui, A., Aubin, I., Berteaux, D., Butler, A., *et al.* (2016). Cross-scale integration of knowledge for predicting species ranges: A metamodelling framework. *Glob. Ecol. Biogeogr.*, 25, 238–249.

Thompson, P.L. & Gonzalez, A. (2017). Dispersal governs the reorganization of ecological networks under environmental change. *Nat. Ecol. Evol.*, 1, 0162.

Torossian, J.L., Kordas, R.L. & Helmuth, B. (2016). *Cross-Scale Approaches to Forecasting Biogeographic Responses to Climate Change*. *Adv. Ecol. Res.* 1st edn. Elsevier Ltd.

Wisz, M.S., Pottier, J., Kissling, W.D., Pellissier, L., Lenoir, J., Damgaard, C.F., *et al.* (2013). The role of biotic interactions in shaping distributions and realised assemblages of species: implications for species distribution modelling. *Biol. Rev.*, 88, 15–30.

Zurell, D., Thuiller, W., Pagel, J., Cabral, J.S., Münkemüller, T., Gravel, D., *et al.* (2016). Benchmarking novel approaches for modelling species range dynamics. *Glob. Chang. Biol.*, 22, 2651–2664.
